# Direct‐stick embolization to treat hematochezia secondary to rectally metastatic sacral neuroblastoma

**DOI:** 10.1002/jpr3.70160

**Published:** 2026-02-26

**Authors:** Anand Dhatt, Leandro Cardarelli‐Leite, Ravjot Dhatt, Collin Barker, Manraj K. S. Heran

**Affiliations:** ^1^ Department of Radiology University of British Columbia Vancouver General Hospital 899 W 12th Avenue Vancouver V5Z 1M9 British Columbia Canada; ^2^ Department of Radiology, Division of Interventional Radiology University of Western Ontario London Ontario Canada; ^3^ Department of Radiology, Division of Interventional Radiology BC Children's Hospital& Vancouver General Hospital Vancouver British Columbia Canada; ^4^ Department of Pediatrics, Division of Gastroenterology BC Children's Hospital Vancouver British Columbia Canada; ^5^ Department of Radiology, Division of Interventional Radiology and Neuroradiology BC Children's Hospital & Vancouver General Hospital Vancouver British Columbia Canada

**Keywords:** chemoembolization, endoscopy, interventional radiology

## Abstract

Neuroblastoma is a malignant tumor that is typically treated with a combination of surgery, radiation therapy, chemotherapy, and immunotherapy. However, minimally invasive embolization and chemoembolization present promising new treatment modalities. We present the case of a 6‐year‐old patient who initially presented with a massive lower GI bleed due to sacral neuroblastoma metastasis invading the rectum, that was managed via transarterial embolization and transrectal endoscopic‐guided percutaneous nBCA embolization. There was significant tumoral supply from the posterior division of the internal iliac arteries treated with coil, liquid, and particle embolics. Subsequently, under endoscopic guidance, liquid embolization via direct puncture of the rectal metastasis was performed to manage any residual vascularity. Thereafter, the patient's lower GI bleed resolved. Direct puncture tumoral embolization offers a potentially highly effective, yet minimally invasive procedure that warrants further evaluation.

## 1 INTRODUCTION

Neuroblastoma is a highly metastatic, malignant tumor. It is the most common and deadly extracranial solid malignancy in childhood.^1^ Given its high propensity to metastasize, the clinical presentation of neuroblastoma is highly variable, as disease can be localized or diffuse. Frequent sites of distant metastasis are bone and liver. Patients are prognosticated according to age at diagnosis, extent of disease, biologic tumor features, and amplification of N‐MYC oncogene.^1^ Based on prognostic factors, patients are risk stratified, and then are typically treated with a combination of surgery, radiation therapy, chemotherapy, and immunotherapy accordingly.^1^, ^2^

Given the often‐advanced disease stage at presentation, many patients are not suitable for conventional treatment modalities. Thus, in conjunction with conventional treatment modalities, intravascular embolization to reduce tumor vascular supply has been successfully described in patients with neuroblastoma, as well as other malignancies.^3^

This case represents a unique technique of direct transrectal embolization of a rectal tumor in addition to intravascular embolization for the management of gastrointestinal bleeding. This manuscript was prepared following the CARE guidelines.

## 2 CASE REPORT

**Figure 1:**
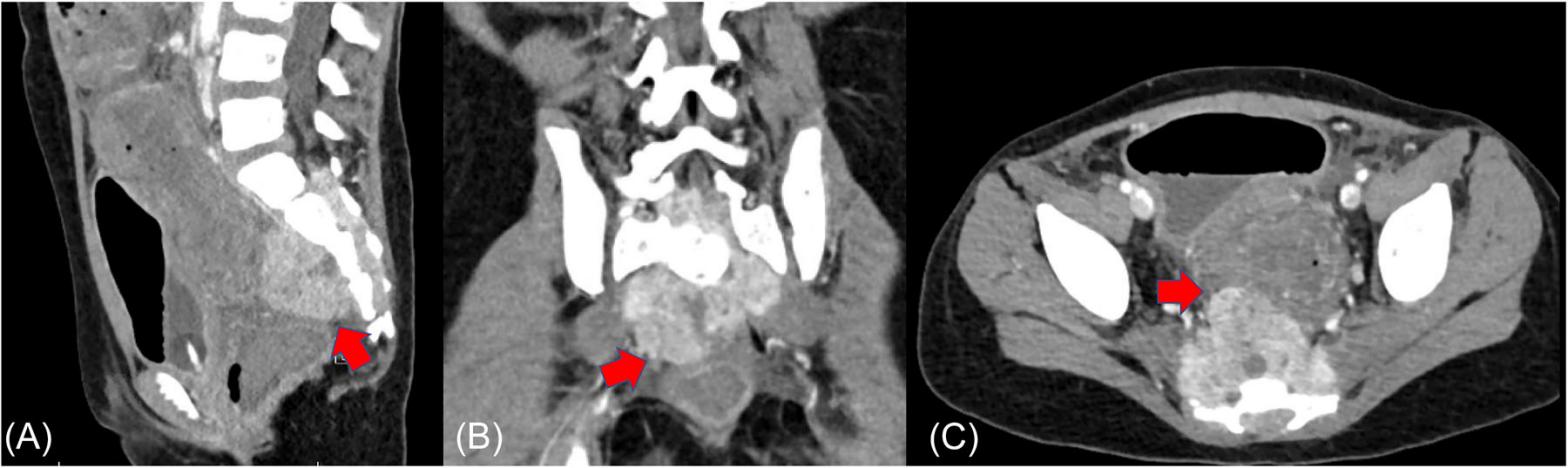
Preprocedural CT demonstrates sacral metastasis (red arrows) with circumferential extraosseous tumor extension into the posterior aspect of the adjacent rectum (yellow arrows). (A) Sagittal. (B) Coronal. (C) Axial. CT, computed tomogra.

The patient was initially diagnosed with stage IV neuroblastoma at 4 years old with metastatic lesions in the bones and liver seen on imaging. Lymph node biopsy at the time of diagnosis demonstrated undifferentiated neuroblastoma with a high Mitotic‐Karyorrhectic Index suggestive of an unfavorable histology. Tumor cytogenetics showed that N‐MYC was not amplified. The patient was initially managed with six cycles of chemotherapy, tumor debulking surgery, and two stem cell transplants. However, later had evidence of relapse for which they began salvage chemoimmunotherapy which was unsuccessful. Subsequently, the patient was transitioned to palliative chemotherapy and then developed recurrent, significant, lower gastrointestinal (GI) hemorrhage, which was refractory to medical therapy. Initial pretreatment computed tomography (CT) scan demonstrated sacral metastasis with circumferential extraosseous tumor extension into the posterior aspect of the adjacent rectum (Figure 1).

### 2.1 Technique

**Figure 2:**
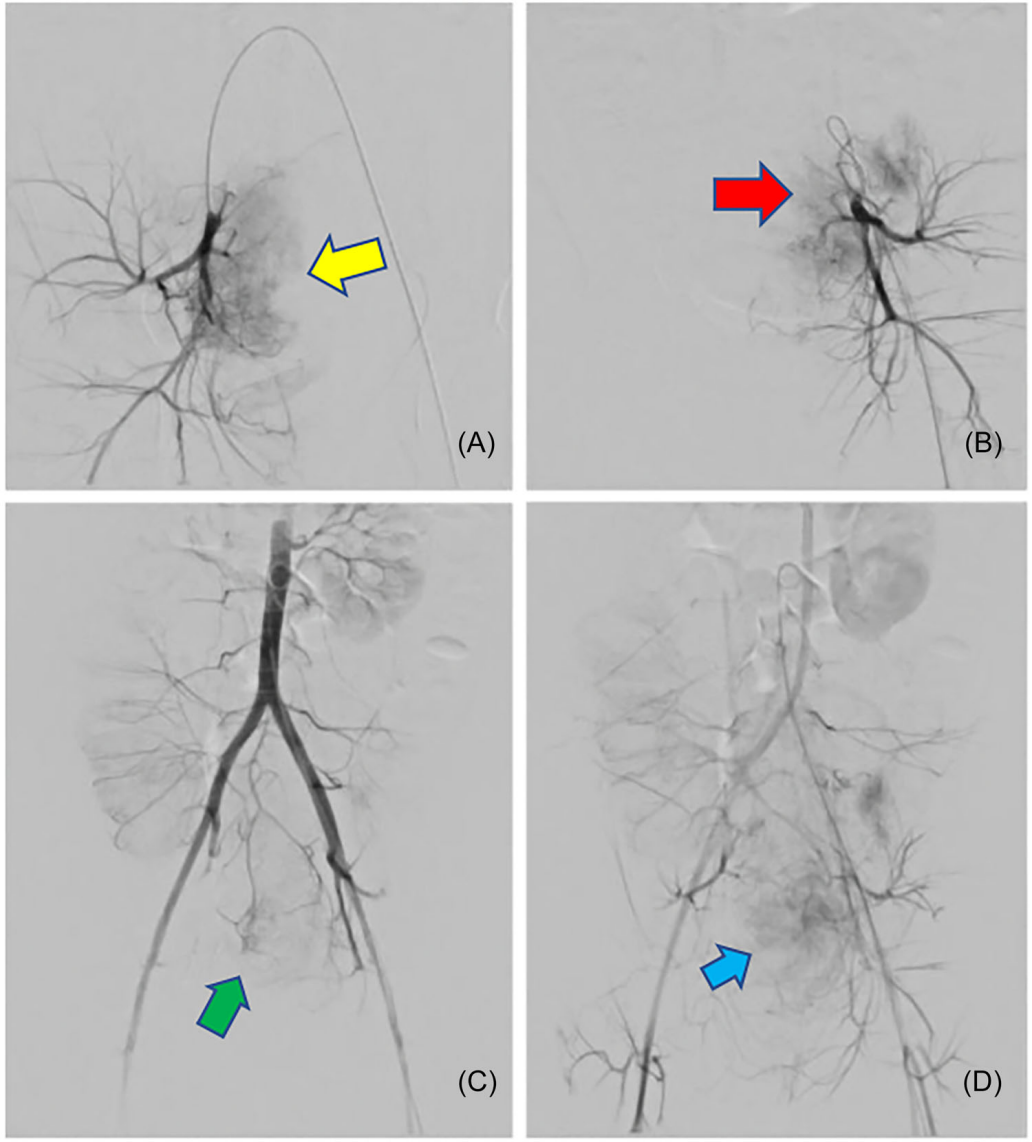
(A) Right IIA angiogram demonstrating significant tumoral blush (yellow arrow), supplying approximately 90% of the tumor. (B) Repeat abdominal aortogram demonstrating 90% devascularization of the tumor (red arrow). (C, D) Residual 10% of tumoral supply from the inferior mesenteric artery (IMA) (green arrow) and left IIA (blue arrow). IIA, internal iliac artery.

The procedure was completed in the angiography suite under general anesthesia. Arterial access was obtained via the right common femoral artery. Initial right internal iliac artery (IIA) angiogram demonstrated significant tumoral blush, supplying approximately 90% of the tumor (Figure 2A). Subsequently, coil embolization was performed of the vesical, rectal, and pudendal branches, followed by particle embolization of the tumor. Additional gel foam slurry was performed of the right IIA. Repeat abdominal aortogram demonstrated 90% devascularization of the tumor (Figure 2B). Residual 10% of tumoral supply was primarily from the inferior mesenteric artery (IMA) (Figure 2C,D), due to risk of causing bowel ischemia, further embolization of the residual tumoral supply was not pursued transarterially.

**Figure 3:**
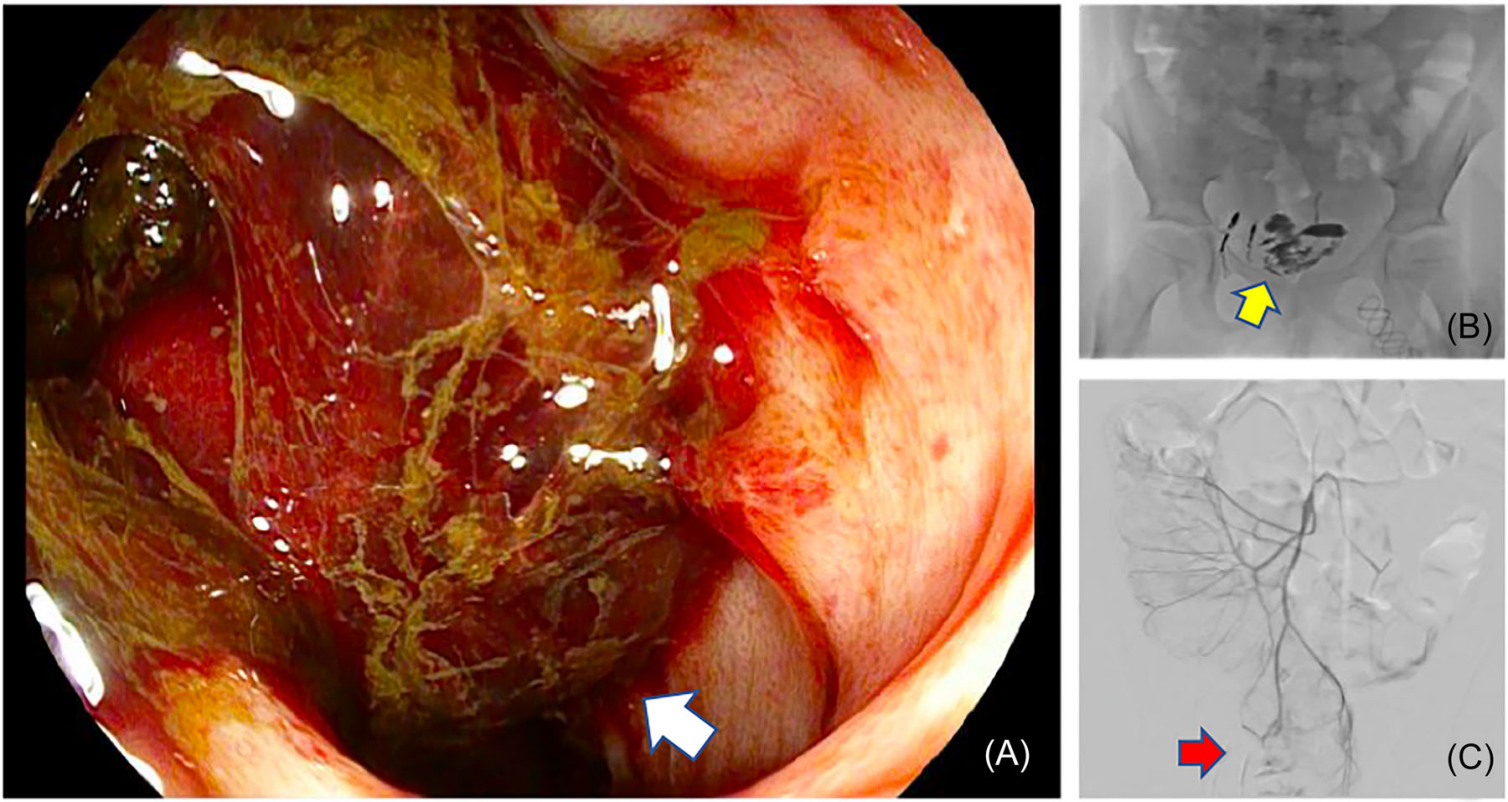
(A) Endoscopy demonstrating a pedunculated submucosal lesion (white arrow). (B, C) Post‐trans‐arterial and direct stick embolization demonstrating reduced tumor blush from the IMA in the area where N‐butyl‐2‐cyanoacrylate (nBCA) embolization was performed (yellow and red arrows).

Subsequently, endoscopy was performed by the GI Service, which allowed direct visualization of the metastasis in the rectum, which appeared as a pedunculated submucosal mass (Figure 3A). The transrectal procedure involved placing a 4 French vascular dilator with its hub cut off immediately adjacent to the endoscope directed towards the tumor. Through this, a 22‐gauge 5” spinal needle with a small angle placed at its tip was advanced and positioned within the submucosal tumor. Glue embolization was then performed with 33% N‐butyl‐2‐cyanoacrylate (nBCA):lipiodol mixture.

Post transrectal embolization, re‐check angiogram demonstrated markedly reduced tumor blush from the IMA in the area where nBCA embolization was performed (Figure 3B,C).

Postprocedurally, the patient had no further GI hemorrhage. There was notably marked reduction of tumor vascularity, enhancement, and size in 14‐day postembolization CT. The patient unfortunately passed away 3 months after the first treatment due to nonbleeding related complications of their stage IV neuroblastoma.

## 3 DISCUSSION

Transcatheter arterial chemoembolization is an effective means of neo‐adjuvant or primary treatment of unresectable tumors of various origins.^4^, ^5^, ^6^ In regard to neuroblastomas in pediatric populations, presurgical transcatheter arterial embolization has been shown to reduce tumor size, intraoperative bleeding, and decrease morbidity, however, the majority of evidence surrounding the use of transcatheter embolization is via case reports.^3^, ^6^ In this case, the choice to initially utilize coil embolization in the vesical, rectal and pudendal branches of the IIA and particle embolization of the tumor were utilized as they result in precise, complete occlusion of high‐flow vessels.^7^ Furthermore, the adjunctive use of gel foam slurry provides temporary embolization of the proximal IIA to further decrease risk of tumoral GI hemorrhage without permanently compromising the IIA.^7^

Due to the difficulty of endovascular access or risk of collateral vessel recruitment in the case of highly vascular lesions, transcatheter embolization is sometimes deferred in favor of direct puncture and localized delivery of embolic material.^8^ Direct puncture embolization is a treatment modality that is predominantly used in the management of vascular lesions and is advantageous over endovascular embolization as it bypasses complex vasculature, mitigates risk of vascular hemorrhage, ischemia, and nontarget embolization.^9^, ^10^

In the case presented, the patient presented with bleeding secondary to rectal metastases of stage IV neuroblastoma. Due to the nature of the patient's disease, while endovascular treatment was able to devascularize 90% of the tumor, it would have potentially been insufficient to halt ongoing hematochezia given the persisting parasitized tumoral blush from the IMA. Additionally, by potentially pursuing intraarterial embolization of the IMA and its branches, there was the potential risk of causing bowel ischemia. Therefore, for this patient the decision to adopt a direct puncture approach for residual embolization was made.

There is no literature surrounding direct puncture embolization of malignant colorectal lesions.

The embolic agents used in direct‐puncture embolization are variable, with literature demonstrating the use of nBCA, polyvinyl alcohol, or Onyx.^10^ In this case, nBCA was utilized as the embolic agent due to easier fluoroscopic visibility, less periprocedural pain, and operator control of viscosity admixed with lipiodol; additionally, the rapid solidification and ability to flow into complex angioarchitecture would allow it to penetrate and embolize the deeper vasculature of the tumor.^7^

## 4 CONCLUSION

This case represents a complex presentation in which a 6‐year‐old patient with advanced stage IV neuroblastoma presented with recurrent significant lower GI hemorrhage, which was successfully treated with transcatheter arterial embolization and novel direct‐puncture embolization to colonic metastases. Direct puncture tumoral embolization represents a potentially highly effective, yet minimally invasive procedure that warrants further evaluation.

## CONFLICT OF INTEREST STATEMENT

The authors declare no conflicts of interest.

## ETHICS STATEMENT

Informed consent for the publication of this case has been obtained from the patient's parents.
